# Response of the Cholesterol Metabolism to a Negative Energy Balance in Dairy Cows Depends on the Lactational Stage

**DOI:** 10.1371/journal.pone.0121956

**Published:** 2015-06-02

**Authors:** Josef J. Gross, Evelyne C. Kessler, Christiane Albrecht, Rupert M. Bruckmaier

**Affiliations:** 1 Veterinary Physiology, Vetsuisse Faculty University of Bern, Bern, Switzerland; 2 Institute of Biochemistry and Molecular Medicine, University of Bern, Bern, Switzerland; 3 Swiss National Center of Competence in Research, NCCR TransCure, University of Bern, Bern, Switzerland

## Abstract

The response of cholesterol metabolism to a negative energy balance (NEB) induced by feed restriction for 3 weeks starting at 100 days in milk (DIM) compared to the physiologically occurring NEB in week 1 postpartum (p.p.) was investigated in 50 dairy cows (25 control (CON) and 25 feed-restricted (RES)). Blood samples, liver biopsies and milk samples were taken in week 1 p.p., and in weeks 0 and 3 of feed restriction. Plasma concentrations of total cholesterol (C), phospholipids (PL), triglycerides (TAG), very low density lipoprotein-cholesterol (VLDL-C) and low density lipoprotein-cholesterol (LDL-C) increased in RES cows from week 0 to 3 during feed restriction and were higher in week 3 compared to CON cows. In contrast, during the physiologically occurring NEB in week 1 p.p., C, PL, TAG and lipoprotein concentrations were at a minimum. Plasma phospholipid transfer protein (PLTP) and lecithin:cholesterol acyltransferase (LCAT) activities did not differ between week 0 and 3 for both groups, whereas during NEB in week 1 p.p. PLTP activity was increased and LCAT activity was decreased. Milk C concentration was not affected by feed restriction in both groups, whereas milk C mass was decreased in week 3 for RES cows. In comparison, C concentration and mass in milk were elevated in week 1 p.p. Hepatic mRNA abundance of *sterol regulatory element-binding factor-2* (*SREBF-2*), *3-hydroxy-3-methylglutaryl-coenzyme A synthase 1* (*HMGCS1*), *3-hydroxy-3-methylglutaryl-coenzyme A reductase* (*HMGCR*), and *ATP-binding cassette transporter* (*ABCA1*) were similar in CON and RES cows during feed restriction, but were upregulated during NEB in week 1 p.p. compared to the non-lactating stage without a NEB. In conclusion, cholesterol metabolism in dairy cows is affected by nutrient and energy deficiency depending on the stage of lactation.

## Introduction

The dairy cow undergoes tremendous physiological changes in lipid metabolism during the transition from gestation to lactation [1,2]. Even during ongoing lactation, homeostatic control of metabolism varies markedly depending on the stage of lactation [2]. With the initiation of copious milk production after calving, energy requirements of dairy cows increase distinctly. Since the postpartum pattern of feed intake does not keep up with the requirements, a negative energy balance (NEB) is the consequence, most severe immediately after parturition [1]. As a consequence, body fat reserves are mobilized resulting in elevated free fatty acids (FFA) concentrations in plasma [2]. FFA are taken up by the liver where they are either processed by the β-oxidation pathway or re-esterified to triglycerides (TAG) and exported as very low density lipoproteins (VLDL) [3]. If the TAG synthesis exceeds the TAG export capabilities as VLDL, fatty liver develops [3]. However, at the onset of lactation the secretion of VLDL by the liver is likely overwhelmed although the hepatic expression of genes involved in cholesterol synthesis, which is an important constituent of VLDL, are upregulated [4].

To mediate the nutrient fluxes towards the mammary gland for milk synthesis during early lactation, extensive endocrine changes coordinating homeorhesis are required. In particular, growth hormone concentration is elevated while insulin and IGF-I are low during the period of homeorhetic regulation of nutrient and energy partitioning to the mammary gland [5]. The adaptation of these hormones to a deliberately induced NEB by feed restriction at around 100 days in milk (DIM) were shown to be similar to those in early lactation, albeit at a much lower amplitude [5]. Changes in blood metabolites (FFA, beta-hydroxybutyrate (BHBA), and glucose) during a NEB in later stages of lactation were also reported to be different in their extent compared to the NEB at the onset of lactation [1,6]. The purpose of the present study was to investigate the response of plasma and milk lipids as well as gene expressions related to cholesterol metabolism to a deliberately induced NEB by feed restriction in mid-lactation. We hypothesized that the adaptation of the cholesterol metabolism to a NEB at around 100 days in milk is physiologically distinct from responses occurring during the early lactational NEB.

## Material and Methods

### Animal trial

The animal trial was approved by the Bavarian state department for animal welfare affairs (permit no. Az. 55.2-1-54-2531-21-08). It was conducted at the Agricultural Experimental Unit Hirschau of the Technical University of Munich, Germany.

Fifty multiparous Holstein dairy cows were studied during 2 periods with a NEB: Directly after parturition during the lactational NEB (week 1 postpartum (p.p.)) and after around 100 DIM (week 14 p.p.) during a period of feed restriction for 3 weeks. Week 0 was designated as the week before initiation of feed restriction, where all cows were in a positive energy balance [1] and split into two groups [25 control cows (CON cows) and 25 restricted cows (RES cows)] following their NEB and milk yield in early lactation. The feeding regimen of CON cows remained unchanged throughout the study, while the RES cows experienced an energy deficiency of approximately 50% of their calculated requirements. CON cows were fed once daily at 0930 h the diet consisting of 6.5% hay, 14.9% concentrate, 33.7% grass silage and 44.9% corn silage (6.53 ± 0.08 MJ NE_L_/kg; crude fat content 32 ± 6 g/kg; dry matter basis) for ad libitum intake as during the previous weeks. Additionally, CON cows received concentrate in transponder access feeding stations (7.96 ± 0.04 MJ NE_L_/kg; crude fat content 24 ± 6 g/kg; 14.9% barley, 24.8% corn kernels, 21.8% wheat, 20.1% soybean meal, 15.2% dried sugar beet pulp with molasses, 3.2% limestone and vitamin-mineral-premix; dry matter basis) according to their milk production. To achieve the intended energy deficiency, RES cows were fed a similar diet as CON cows, but mixed with more hay to reduce its energy content (6.24 ± 0.05 MJ NE_L_/kg; crude fat content 28 ± 6 g/kg; 21.8% grass silage, 29.1% corn silage, 9.7% concentrate, 39.4% hay; dry matter basis) and a limited amount of concentrate (0.4 kg/d; dry matter basis).

### Blood and milk sampling

Cows were bled from the jugular vein between 0730 h and 0900 h before feeding in week 1 p.p., and in week 0 and 3 of the feed restriction period. The obtained blood samples were immediately cooled down on wet ice, centrifuged for 15 min at 2,000 x *g*, and the plasma was stored at -20°C until analysis.

Milk sampling was performed once per week at 0500 h in week 1 p.p., week 0 and 3 of the feed restriction period. Milk samples were stored at -20°C until analysis.

### Plasma lipids

Plasma concentrations of total cholesterol (C) and TAG were measured with enzymatic kits no. 61219 for C and no. 61236 for TAG (both from bioMérieux SA, Geneva, Switzerland). PL and free cholesterol (FC) concentrations were measured using enzymatic kits from Wako Pure Chemical Industries Ltd., Osaka, Japan (no. 296–63801 for PL; no. 435–35801 for FC). High density lipoprotein-cholesterol (HDL-C) was measured using an immunoinhibition method (kit no. 412–72395) and LDL-C with an enzymatic kit (no. 419–24017) (both from Wako Chemical GmbH, Neuss, Germany).

Concentrations of cholesteryl esters (CE) and VLDL-C were calculated according the following equations: [7]

Cholesteryl esters=cholesterol−free cholesterol;

VLDL-C=TAG/5

### Plasma enzyme activities

The plasma activities of lecithin:cholesterol acyltransferase (LCAT) and phospholipid transfer protein (PLTP) were measured with fluorescence activity assays (no. RB-LCAT for LCAT, no. P7700 for PLTP; Roar Biomedical, New York, USA) according to the manufacturer’s instructions. LCAT activity is expressed as the ratio of the emissions of the substrate hydrolyzed and not hydrolyzed.

### Milk cholesterol

Cholesterol was extracted from raw milk according to modified protocols of Paradkar and Irudayaraj [8] and Kamelska et al. [9] and then measured with an enzymatic kit (no. 61219; bioMérieux SA, Geneva, Switzerland). The method is explained in detail in Kessler et al. [4].

### Milk fat content

Milk fat concentrations were measured by an infrared analyzer (MilkoScan FT-6000, Foss Analytical A/S, Hillerød, Denmark; Milchpruefring Bayern e.V., Wolnzach, Germany).

### Liver tissue sampling and hepatic gene expressions

Liver tissue samples were taken in parallel with blood samples by blind percutaneous needle biopsy in week 1 p.p., and in week 0 and 3 of the feed restriction period as described by Gross et al. [5]. Thereafter, liver tissue was put into an RNA stabilization reagent (RNAlater; Ambion, Applied Biosystems, Austin, TX, USA), kept at +4°C for 24 h, and then stored at -20°C until analysis. For isolation of RNA, peqGOLD TriFast (PEQLAB Biotechnologie GmbH, Erlangen, Germany) was used. RNA integrity was verified by the optical density (OD) ratio of 260 and 280 nm, which was between 1.7 and 2.1 for all samples. After reverse transcription of 1 *μ*g of total RNA into cDNA, PCR quantification of the ATP-binding cassette transporter (*ABC*) *A1*, *ABCG1*, 3-hydroxy-3-methylglutaryl-coenzyme A reductase (*HMGCR*), 3-hydroxy-3-methylglutaryl-coenzyme A synthase 1 (*HMGCS1*), liver X receptor α (*LXRα*), microsomal triglyceride transfer protein (*MTTP*), peroxisome proliferator activated receptor (*PPAR*) *α* and *γ*, sterol regulatory element-binding factor (*SREBF*) *-1* and—*2* genes was performed. Since the hepatic expression of glyceraldehyde 3-phosphate dehydrogenase (*GAPDH*) and *ubiquitin* was stable during the entire experiment, they were used as reference genes and the mRNA abundance of the target genes was calculated relative to them. Details about the primers for the measured genes and methods used are given in Table 1 and described in Gross et al. [5] and Kessler et al. [4].

**Table 1:** Sequence of forward and reverse primers for real-time PCR.

Gene^1^	Gene accession no.		Sequence (5’-3’)
*ABCA1*	NM_001024693	Forward	GGACATGTGCAACTACGTGG
		Reverse	TGATGGACCACCCATACAGC
*ABCG1*	XM_587930	Forward	GACTCGGTCCTCACGCAC
		Reverse	CGGAGAAACACGCTCATCTC
*GAPDH*	NM 001034034	Forward	TACATGGTCTACATGTTCCAGTATG
		Reverse	CAGTCTTCTGGGTGGCAGTGATG
*HMGCR*	BC153262	Forward	ACCCATGAGCGAGGTGTATC
		Reverse	TAGTGCTGGCCACAAGACAG
*HMGCS1*	BC102850	Forward	TGTACGGCTCCCTGGCTTCTG
		Reverse	CATGTTCCTTCGAAGAGGGAATC
*LXRα*	NM_001014861.1	Forward	CTGCGATTGAGTGATGCTC
		Reverse	CGGTCTGCAGAGAAGATGC
*MTTP*	NM_001101834.1	Forward	TCATCCAATGTGGATGTCGC
		Reverse	GAGATTTTCTATGGCTGCTG
*PPARα*	NM_001034036.1	Forward	AGGGCTGCAAGGGTTTCTTTAG
		Reverse	TGACGAAAGGCGGGTTGTTGTTG
*PPARγ*	NM_181024.2	Forward	AACTCCCTCATGGCCATTGAATG
		Reverse	AGGTCAGCAGACTCTGGGTTC
*SREBF1*	NM_001113302.1	Forward	CCAGCTGACAGCTCCATTGA
		Reverse	TGCGCGCCACAAGGA
*SREBF2*	XM_583656.3	Forward	GACTGATGCCAAGATGCACA
		Reverse	CCCTTCAGGAGTTTGCTCTT
*UBQ*	Z18245	Forward	AGATCCAGGATAAGGAAGGCAT
		Reverse	GCTCCACTTCCAGGGTGAT

^1^ *ABCA1*: ATP-binding cassette transporter A1; *ABCG1*: ATP-binding cassette transporter G1; *GAPDH*: glycerinaldehyd-3-phosphat-Dehydrogenase; HMGCR: 3-hydroxy-3-methylglutaryl-coenzyme A reductase; *HMGCS1*: 3-hydroxy-3-methylglutaryl-coenzyme A synthase 1; *LXRα*: liver X receptor α; *MTTP*: microsomal triglyceride transfer protein; *PPARα*: peroxisome proliferator activated receptor α; *PPARγ*: peroxisome proliferator activated receptor γ; *SREBF-1*: sterol regulatory element-binding factor 1; *SBREF-2*: sterol regulatory element-binding factor 2; *UBQ*: ubiquitin.

### Statistical analysis

Data presented in the text and tables are means ± standard error of the mean. Changes in blood parameters, milk cholesterol and hepatic mRNA abundances between week 1 p.p. and week 3 in RES cows as well as the effect of feed restriction between week 0 to 3 for both groups were evaluated with a MIXED model including group as fixed effect. Furthermore, concentrations of blood parameters, milk cholesterol and hepatic mRNA abundance of week 0 were used as co-variable and the individual cow as repeated subject. Differences between groups were detected by the Bonferroni t-test. *P-*values < 0.05 were considered to be significant.

## Results

**Fig 1:**
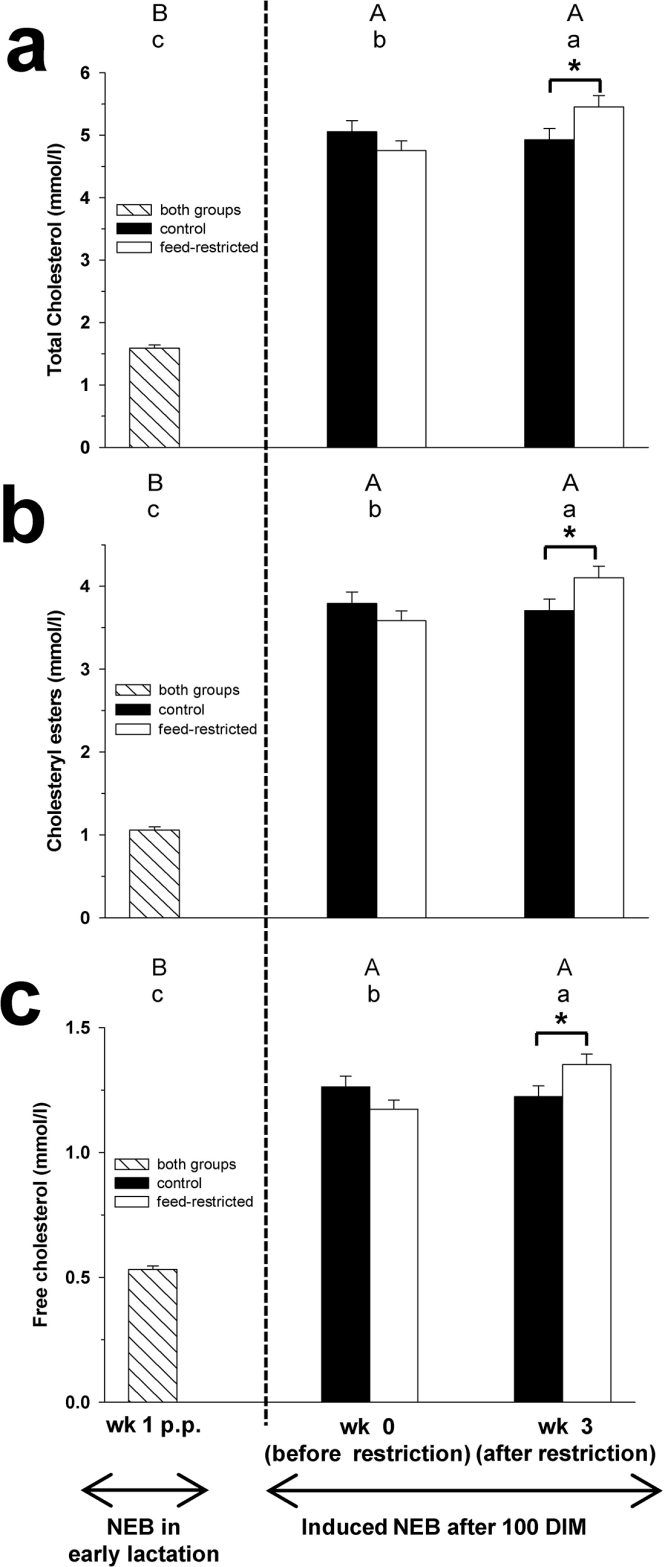
Total cholesterol, cholesteryl esters and free cholesterol. Plasma concentration of total cholesterol (Fig 1a), cholesteryl esters (Fig 1b) and free cholesterol (Fig 1c) in week 1 p.p., and before (week 0) and after (week 3) feed restriction in mid-lactation. Changes over time within the groups are marked with superscripts (A to C for the control group (CON); a to c for the feed-restricted group (RES); *P* < 0.05). Differences between control (CON) and feed-restricted (RES) group in week 3 are indicated by * (*P* < 0.05). Data are presented as means ± standard error of the mean.

**Fig 2:**
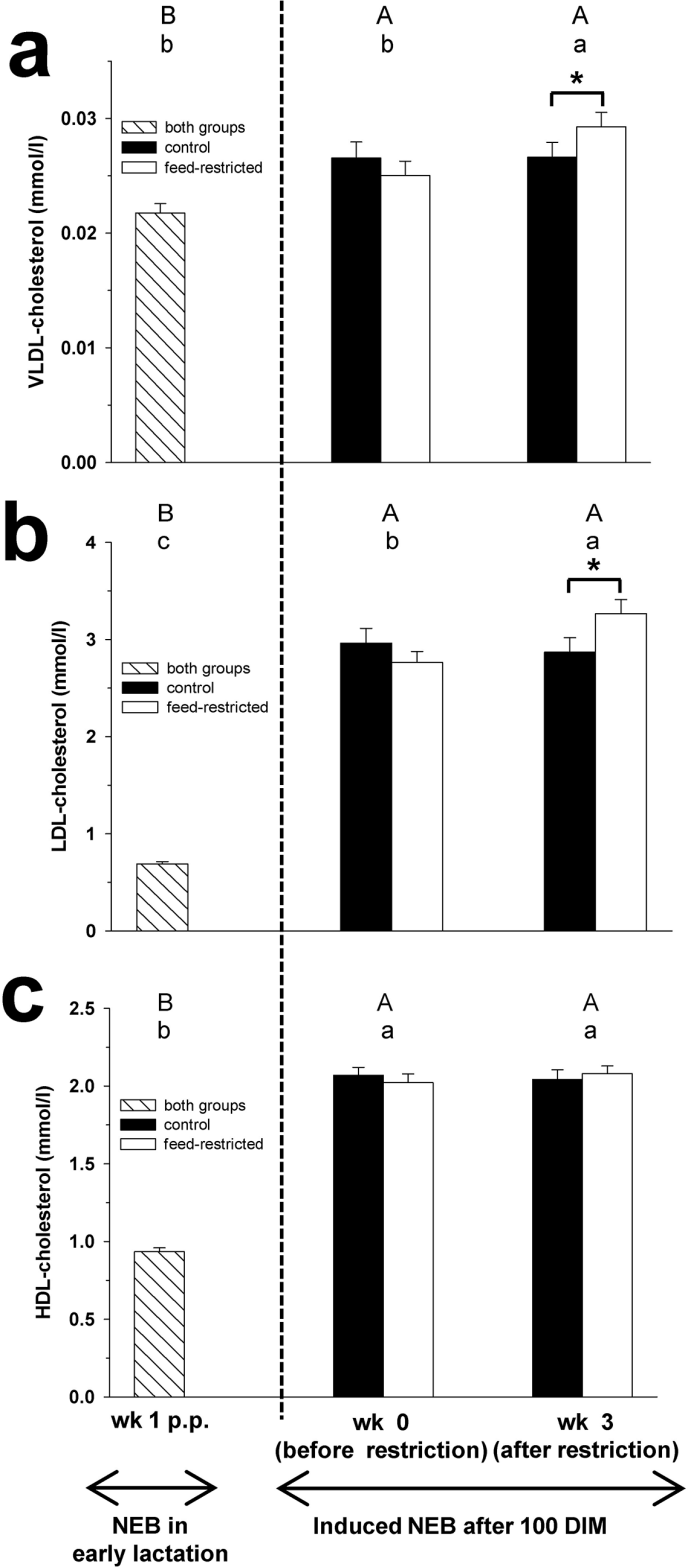
VLDL-cholesterol, LDL-cholesterol and HDL-cholesterol. Plasma concentration of VLDL-cholesterol (Fig 2a), LDL-cholesterol (Fig 2b) and HDL-cholesterol (Fig 2c) in week 1 p.p., and before (week 0) and after (week 3) feed restriction in mid-lactation. Changes over time within the groups are marked with superscripts (A to C for the control group (CON); a to c for the feed-restricted group (RES); *P* < 0.05). Differences between control (CON) and feed-restricted (RES) group in week 3 are indicated by * (*P* < 0.05). Data are presented as means ± standard error of the mean.

**Fig 3:**
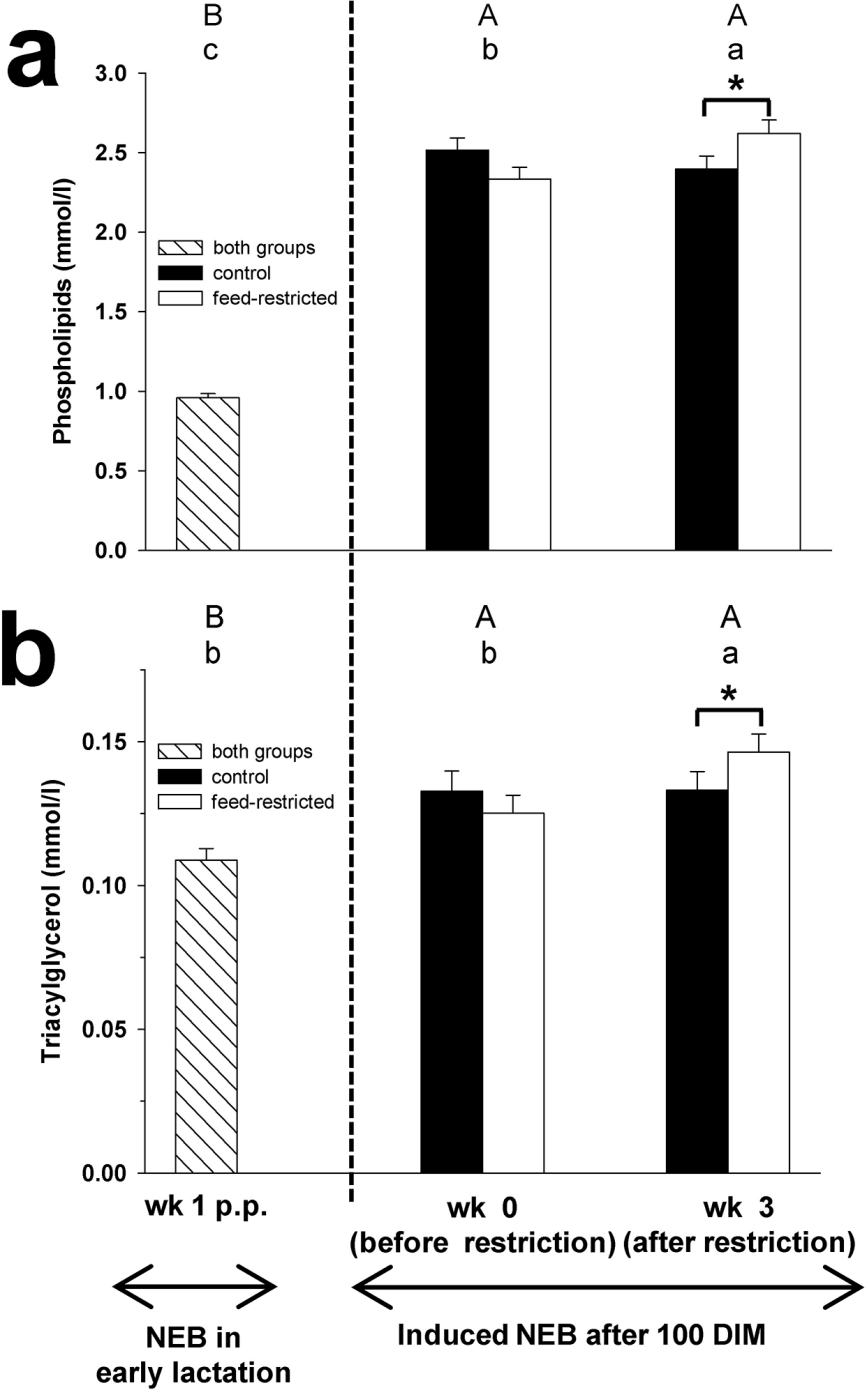
Phospholipids and TAG. Plasma concentration of phospholipids (Fig 3a) and triacylgycerol (Fig 3b) in week 1 p.p., and before (week 0) and after (week 3) feed restriction in mid-lactation. Changes over time within the groups are marked with superscripts (A to C for the control group (CON); a to c for the feed-restricted group (RES); *P* < 0.05). Differences between control (CON) and feed-restricted (RES) group in week 3 are indicated by * (*P* < 0.05). Data are presented as means ± standard error of the mean.

**Fig 4:**
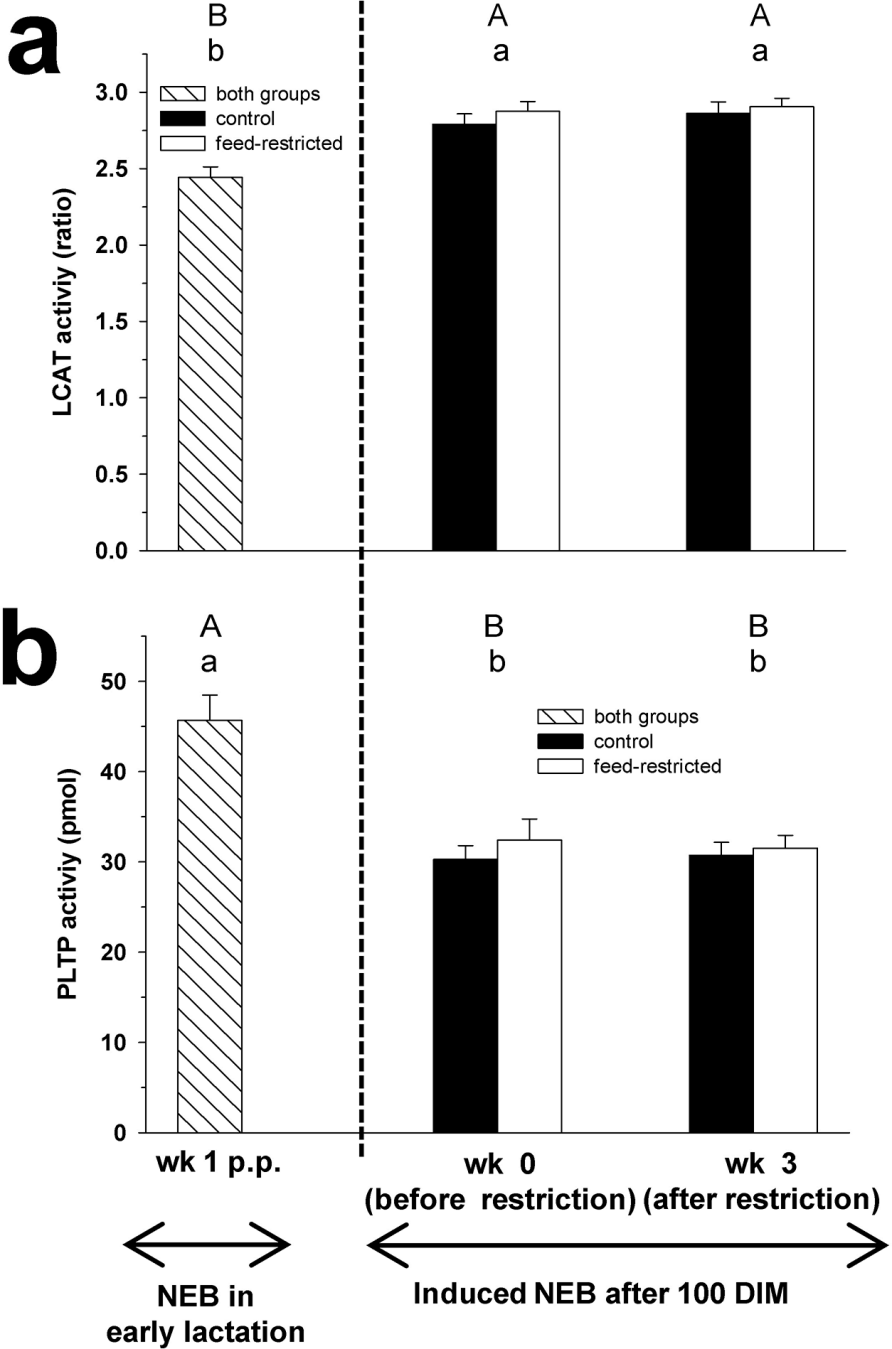
LCAT and PLTP activities. Plasma activity of lecithin:cholesterol acyltranserase (LCAT; Fig 4a) and phospholipid transfer protein (PLPT; Fig 4b) in week 1 p.p., and before (week 0) and after (week 3) feed restriction in mid-lactation. Changes over time within the groups are marked with superscripts (A and B for the control group (CON); a and b for the feed-restricted group (RES); *P* < 0.05). Data are presented as means ± standard error of the mean.

**Fig 5:**
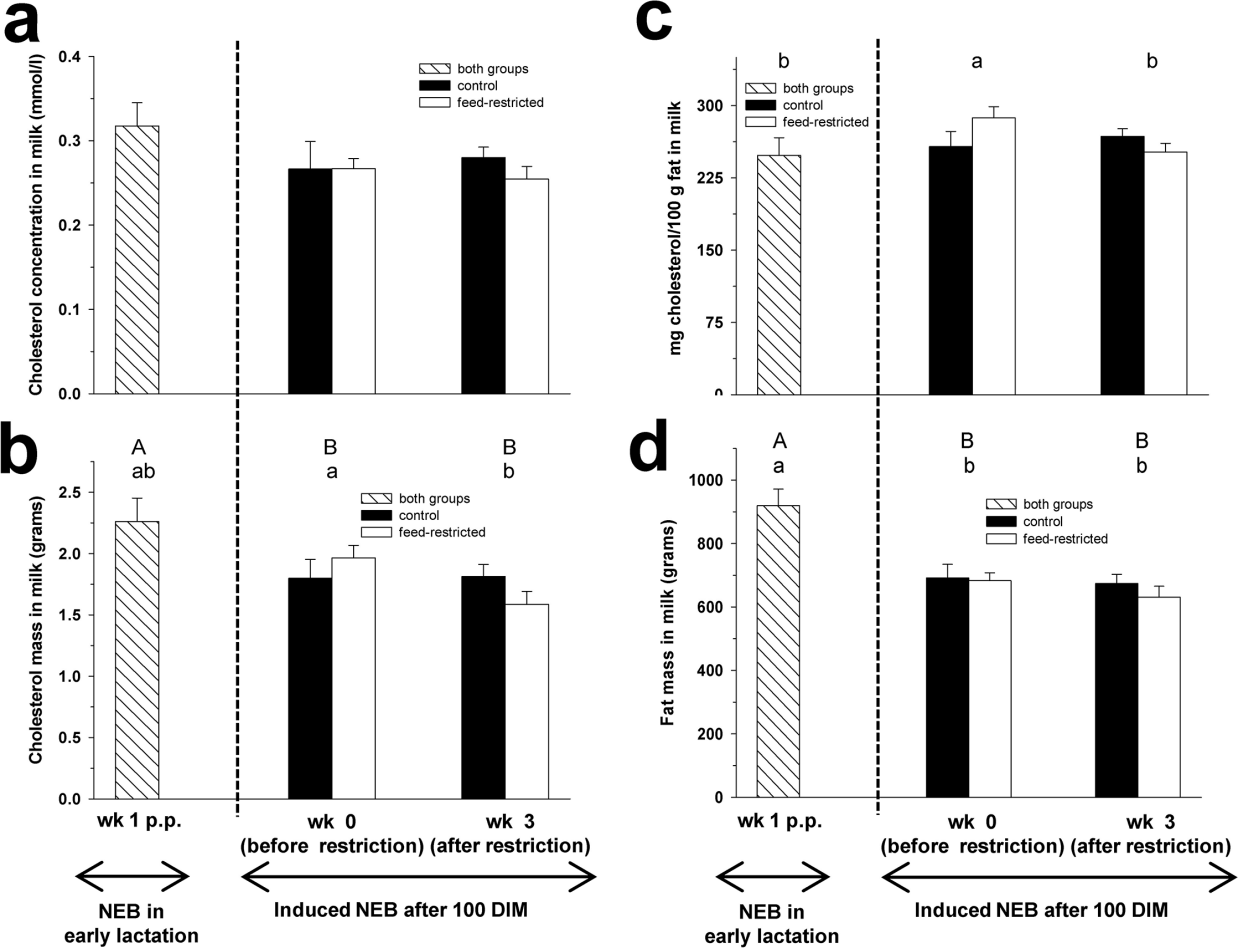
Milk cholesterol. Cholesterol concentration (mmol/l) in milk (Fig 5a), cholesterol mass (grams) in milk (Fig 5b), cholesterol concentration in milk fat (mg cholesterol per 100 g fat; Fig 5c) and fat mass (grams) in milk (Fig 5d) in week 1 p.p., and before (week 0) and after (week 3) feed restriction in mid-lactation. Changes over time within the groups are marked with superscripts (A and B for the control group (CON); a and b for the feed-restricted group (RES); *P* < 0.05). Data are presented as means ± standard error of the mean.

The concentrations of plasma lipids, enzyme activities, hepatic gene expressions and milk cholesterol in week 1 p.p. presented in Figs 1, 2, 3, 4 and 5 and Table 2 have already been published elsewhere [4]. These data were added to the tables to provide a better overview and to simplify the comparison between the NEB induced by feed restriction in mid-lactation and the physiological NEB caused by the initiation of lactation after parturition.

**Table 2:** Relative mRNA abundances (delta CT, log_2_) of genes analyzed in liver biopsies in week 1 p.p., and before (week 0) and after (week 3) feed restriction in mid-lactation.

	Lactational NEB	Induced NEB by feed restriction after 100 DIM			
	week 1 p.p.	week 0		week 3	
	both groups	CON cows	RES cows	CON cows	RES cows
Parameter	(n = 50)	(n = 25)	(n = 25)	(n = 25)	(n = 25)
Cholesterol synthesis					
*HMGCR*	18.12 ± 0.26	18.01 ± 0.31	17.59 ± 0.37	18.24 ± 0.26	18.11 ± 0.37
*HMGCS1*	19.31 ± 0.03	19.06 ± 0.30	18.68 ± 0.33	19.38 ± 0.29	18.68 ± 0.46
VLDL assembly					
*MTTP*	17.03 ± 0.15	16.86 ± 0.19	16.71 ± 0.23	16.80 ± 0.24	16.92 ± 0.28
Cholesterol transport					
*ABCA1*	17.26 ± 0.25^a^	17.20 ± 0.26	16.30 ± 037^b^	17.23 ± 0.27	16.46 ± 0.37^ab^
*ABCG1*	10.68 ± 0.22	11.34 ± 0.19	11.18 ± 0.34	11.05 ± 0.27	11.69 ± 0.26
Nuclear receptors					
*LXRα*	16.53 ± 0.14	16.46 ± 0.14	16.50 ± 0.17	16.51 ± 0.14	16.47 ± 0.17
*PPARα*	17.35 ± 0.14	17.31 ± 0.18	17.46 ± 0.18	17.40 ± 0.19	17.48 ± 0.19
*PPARγ*	10.73 ± 0.14	10.63 ± 0.16	11.03 ± 0.24	10.53 ± 0.23	10.77 ± 0.23
Other regulatory genes					
*SREBF-1*	13.64 ± 0.14^Bb^	14.47 ± 0.16^A^	14.38 ± 0.22^a^	14.62 ± 0.17^A^	14.37 ± 0.23^a^
*SREBF-2*	17.83 ± 0.23	17.77 ± 0.19	17.18 ± 0.41	17.76 ± 0.23	17.53 ± 0.32

Changes over time within the groups are marked with superscripts (A and B for the control group (CON); a and b for the feed-restricted group (RES); *P* < 0.05).

Hepatic mRNA expression was analyzed in week 1 p.p., week 0 and week 3. Data are presented as means ± standard error of the mean. Abbreviations: *ABCA1*: ATP-binding cassette transporter A1; *ABCG1*: ATP-binding cassette transporter G1; CON: control cows; DIM: days in milk; HMGCR: 3-hydroxy-3-methylglutaryl-coenzyme A reductase; *HMGCS1*: 3-hydroxy-3-methylglutaryl-coenzyme A synthase 1; *LXRα*: liver X receptor α; *MTTP*: microsomal triglyceride transfer protein; NEB: negative energy balance; *PPARα*: peroxisome proliferator activated receptor α; *PPARγ*: peroxisome proliferator activated receptor γ; RES: feed-restricted cows; SREBF-*1*: sterol regulatory element-binding factor 1; *SBREF-2*: sterol regulatory element-binding factor 2.

### Plasma lipids

The plasma concentrations of C, CE, FC, VLDL-C, LDL-C, PL and TAG increased during feed restriction from week 0 to 3 in RES cows, whereas in CON cows no changes were found (Figs 1, 2 and 3). In week 3 of feed restriction, the concentrations of these parameters were higher in RES cows compared to CON cows (*P* < 0.05).

The plasma concentration of HDL-C did not differ between week 0 and week 3 for both groups (Fig 2C). In week 3, no differences in HDL-C concentration between the groups were observed.

Plasma concentrations of C, CE, FC, VLDL-C, LDL-C, HDL-C, PL and TAG in CON and RES cows were higher in week 3 of feed restriction compared to week 1 p.p. (Figs 1, 2 and 3).

### Plasma enzyme activities

During feed restriction, the plasma LCAT and PLTP activities did not change between week 0 to week 3 for both groups (Fig 4). No differences in LCAT and PLTP activities were found between groups in week 3 of feed restriction.

The activity of LCAT in plasma in CON and RES cows was higher in week 3 of feed restriction compared to week 1 p.p.. In contrast, the PLTP activity was decreased in week 3 of feed restriction compared to week 1 p.p. in CON and RES cows.

### Milk cholesterol

The concentration of C in milk did not differ between week 0 and 3 for both groups nor between CON and RES cows in week 3 of restriction (Fig 5A). Similarly, the concentration of C in milk in CON and RES cows showed no differences between week 3 of feed restriction and week 1 p.p..

Mass of C in milk decreased during feed restriction from week 0 to 3 in RES cows, while in CON cows no changes were observed (Fig 5B). In week 3 of restriction, cholesterol mass in milk was lower by trend in RES cows compared to CON cows (*P* = 0.06). Cholesterol mass in milk in RES cows did not change between week 3 of feed restriction and week 1 p.p., whereas for CON cows C mass in milk was higher in week 1 p.p. compared to week 3 of feed restriction.

The cholesterol concentration in milk fat (mg cholesterol per 100 g fat) decreased in RES cows (*P* = 0.0473), while in CON cows no differences were found between week 0 and 3 of feed restriction (Fig 5C). In week 3 of feed restriction, mg cholesterol per 100 g fat in milk did not differ between CON and RES cows. The cholesterol content per 100 g fat in milk in CON and RES cows did not change between week 3 of feed restriction and week 1 p.p..

Mass of fat in milk did not change during feed restriction between week 0 and 3 for both groups (Fig 5D). Fat mass in milk showed no differences between CON and RES cows in week 3 of restriction. Fat mass in CON and RES cows was lower in week 3 of feed restriction compared to week 1 p.p. (Fig 5D; *P* = 0.0019).

### Hepatic gene expressions

The hepatic mRNA abundances of *ABCA1* and *ABCG1* did not change between week 0 and 3 of feed restriction for both groups (Table 2). In week 3 of restriction, no differences in hepatic mRNA abundance of *ABCA1* and *ABCG1* were found between CON and RES cows. Expressions of *ABCA1* and *ABCG1* in the liver in CON and RES cows did not change between week 3 of feed restriction and week 1 p.p..

The mRNA expression of *HMGCR*, *HMGCS1*, *SREBF-1* and *SREBF-2* neither showed differences between week 0 and 3 in CON and RES cows nor between groups in week 3 of feed restriction. Similarly, no changes for these genes were observed in CON and RES cows comparing week 3 of feed restriction and week 1 p.p., except for SREBF-1 which was higher in week 3 of feed restriction in both, CON and RES cows, compared to week 1 p.p. (Table 2).

No differences in the mRNA abundances of *MTTP*, *LXRα*, *PPARα* and *PPARγ* were found between any of the tested groups (CON and RES cows between week 0 and 3 during feed restriction; *P* > 0.05; CON and RES cows in week 3 of restriction; *P* > 0.05; CON and RES cows between week 3 of feed restriction and week 1 p.p.; *P* > 0.05; Table 2).

## Discussion

The aim of the present study was to compare the effects of the physiologically occurring NEB in early lactation with a deliberately induced NEB at later stages of lactation focusing on hepatic lipid metabolism. Thereby a wide variety of lipid-related parameters were measured both in plasma and in milk along with enzyme activities and hepatic gene expressions of regulators and transporters involved in lipid homeostasis.

After parturition, the nutritional and energetic needs of dairy cows increase considerably. The energy requirements cannot be met through feed intake because the intakes do not increase to the extent needed to support the increase in milk production in early lactation. Simultaneously, distinct endocrine changes affecting predominately glucose and lipid metabolism are triggered to ensure the homeorhetic nutrient partitioning towards the prioritized mammary gland despite a catabolic state [5]. This selectivity in directing nutrients coincides with the reduced responsiveness and sensitivity of extrahepatic tissues to insulin, i.e. insulin resistance, is thought to be markedly involved in developing ketosis and hepatic lipidosis [10]. Insulin is additionally known to suppress lipolysis from adipose tissue [10]. However, plasma insulin concentration in dairy cows is decreased after parturition and enables along with the concomitant insulin resistance and the associated loss of inhibitory effects on lipolysis the high degree of metabolic priority [5]. Consequently, body fat reserves are mobilized and FFA serving as energy source are transported to the liver. During the NEB in early lactation, however, the liver is not able to deal with the excessive FFA supply by both β-oxidation and export of TAG, which results in an accumulation of TAG in the liver [3]. During the deliberately induced NEB in mid-lactation, the plasma FFA concentration was elevated in RES cows in week 3 of restriction, but to a smaller extent than in early lactation [1]. Compared to the high priority after parturition, the importance of nutrient partitioning towards the mammary gland declines continuously and establishes a homeostatic control of metabolism reflected by the restored insulin responsiveness of peripheral tissues and the only moderate and not significant decline in insulin concentration during feed restriction in mid-lactation [5]. Nevertheless, the increased FFA concentration due to feed restriction implies a higher production of TAG in the liver despite an enhanced oxidation capacity at this later stage of lactation. Surprisingly and in contrast to the NEB after parturition, TAG and VLDL-C plasma concentrations increased in RES cows in mid-lactation during a similar severe NEB as cows were exposed to at the onset of lactation. Thus, despite a comparable energy deficiency as in early lactation, the liver seems to be capable of enhancing the secretion of TAG as VLDL to avoid the development of hepatic steatosis in mid-lactation [11]. Fatty liver did not occur in feed-restricted cows of the present study, since the hepatic TAG content was significantly lower in week 3 compared to week 1 p.p. [11].

During the early lactational NEB, the VLDL secretion is impaired despite an upregulation of the rate-controlling key enzymes for cholesterol biosynthesis, HMGCR and HMGCS1, and the sterol synthesis stimulating gene *SREBF-2* [4,12], which is thought to be a limiting factor for the export of VLDL [13]. During the period of feed restriction at a later lactational stage in the present study, however, the mRNA abundances of these genes were not affected. In contrast, in the study of Loor et al. [13] cows experienced a period of feed restriction at the onset of lactation and showed decreased expressions of *SREBF-2*, *HMGCR* and *HMGCS1*. These results demonstrate a different response of the sterol synthetic genes towards an energy deficiency at different stages of lactation, and thus also in different metabolic and endocrine states, and further suggest that in mid-lactation enough cholesterol is available to meet the increasing demand for the formation of VLDL.

Lipoproteins are composed of apoproteins (apo), TAG, cholesterol and PL [14]. During the induced NEB at around 100 DIM, PL and C plasma concentrations were elevated in RES cows, whereas during the NEB at the onset of lactation these parameters were decreased [4]. Bjerre-Harpøth et al. [6] conducted a study with a NEB at different stages of lactation and confirmed the increase in C concentration, but found no changes in PL concentrations. Those authors suggested the elevated plasma C concentrations being a result of the increased secretion of VLDL by the liver. It was also assumed that a decreased hepatic PL synthesis may limit the VLDL secretion [15]. The findings of the present study, however, suggest that during a NEB in mid-lactation, PL are sufficiently available to meet the increased requirements for VLDL synthesis. The effects of insulin on synthesis and degradation of apoB and on the export of TAG as VLDL from hepatocytes reported in literature are controversial [10]. On the one hand insulin was shown to suppress VLDL secretion and apoB synthesis by the liver, while other findings suggest that insulin enhances TAG secretion and reduces degradation of apoB (for review, see [10]).

For the assembly of VLDL, the protein MTTP is required to transfer TAG to apoB [16]. Similarly to the NEB after parturition in the present study, the mRNA abundance of *MTTP* was not significantly altered during the NEB induced by feed restriction. These results are in agreement with a previous study investigating feed restriction at the onset of lactation [13]. Furthermore, the nutritional status was shown not to affect the activity or mass of MTTP [17]. Despite numerous studies related to lipid metabolism in dairy cows, knowledge about the role of insulin and other endocrine factors on limitations in hepatic VLDL export needs to be increased.

After secretion from the liver, VLDL in the plasma are modified to LDL by hydrolysis of the TAG by lipoprotein lipase. LDL are rich in cholesterol and carry the latter to the peripheral tissues. In the present study, RES cows showed an increase of LDL-C plasma concentration during the period of feed restriction. These results are consistent with a fasting-refeeding study conducted in mice which also reported an increased serum LDL-C [18]. This increased LDL-C concentration is likely a consequence of the enhanced export of VLDL from the liver, enabled by the anabolic state of the metabolism, and their subsequent modification to LDL.

FC is collected from extrahepatic tissues by HDL and esterified with lecithin to CE by the enzyme LCAT. During the NEB after parturition, plasma LCAT activity is decreased in cows with fatty liver as well as in healthy cows [4,19,20]. Nakagawa et al. [19] attributed the reduced plasma LCAT activity to the concomitant low apolipoprotein A-I (Apo A-I) concentration which is the activator of the LCAT reaction [22]. Furthermore, Apo A-I is the major apoprotein in HDL [14] and HDL-C was decreased at the onset of lactation [4]. However, during the deliberately induced NEB around 100 DIM, plasma LCAT activity showed no change in RES cows and stayed on the same high level as in CON cows without any insulin resistance. Contrary to the present findings in dairy cows, plasma LCAT activity was shown to be increased in human patients with insulin resistance [21]. Moreover, and contrary to the early lactational NEB, HDL-C plasma concentration was also not affected by the NEB induced by feed restriction and remained elevated. Thus, when lactation is established and homeostatic control of metabolism has taken place, the Apo A-I secretion seems not affected by the NEB and the LCAT activity is not impaired. Furthermore, during the deliberately induced NEB in mid-lactation CE as well as FC increased in RES cows while the LCAT activity remained unchanged. Contrary to the early lactational NEB, the plasma CE concentration during the NEB induced in mid-lactation was decreased due to the reduced plasma LCAT activity. Thus, the increased concentrations of CE and FC in the present study are presumably a consequence of the increased hepatic VLDL secretion and the concomitant increase in LDL since the plasma LCAT activity remained constant.

While the plasma LCAT activity was decreased in week 1 p.p., the PLTP activity was elevated presumably to supply the LCAT reaction with PL for the production of CE [4]. PLTP stimulates the PL transfer from VLDL to HDL [23] and enhances the efflux of cholesterol and PL from tissues by HDL apolipoprotein [24]. However, PLTP activity was not affected by the deliberately induced NEB at 100 DIM in line with the unchanged LCAT activity. Thus, in mid-lactation sufficient PL are available to supply the LCAT reaction and PLTP activity is not triggered. In agreement with our findings, [21] reported that a higher PLTP activity was related to insulin resistance in humans. Therefore, insulin resistance in dairy cows can be considered to affect PLTP activity.

High levels of free cholesterol are toxic to cells. To avoid excessive cholesterol accumulation, cells have a sensor of cholesterol levels, LXRα, which is activated by oxysterols [25]. The efflux of cholesterol across cellular membranes is mediated by ABCA1 [26] and ABCG1 which cooperate to increase cholesterol export to apo A-I and HDL, respectively [27]. ABCA1 and ABCG1 are controlled by LXRα as well as other regulatory processes. The NEB induced by feed restriction did neither affect the expression of *ABCA1* nor *ABCG1* even though their expression pattern was shown to depend on the lactational stage [4,12,28,29]. The lacking effects of insulin resistance on ABCA1 expression was shown previously also for humans [21]. The unchanged expression pattern of these ABC transporters at 100 DIM were accompanied both by an unchanged mRNA abundance of *LXRα* and similar HDL-C plasma concentrations during the deliberately induced NEB emphasizing the different adaptation of dairy cow’s metabolism to a NEB at different stages of lactation.

Schultz et al. [30] postulated that LXRα also induces SREBF-1 and thus activates lipogenic genes like acetyl-CoA carboxylase (ACC) or fatty acid synthase (FASN). In the present study, feed-restricted cows showed no change of hepatic *SREBF-1* mRNA abundance in week 3, and accordingly the hepatic expression of *FASN* and *ACC* remained also unchanged [11]. In contrast, *SREBF-1* was downregulated during the NEB at the onset of lactation and concomitantly expressions of *ACC* and *FASN* were lower compared to mid-lactation [11,31]. Furthermore, in cows subjected to NEB at the onset of lactation, the target genes *SREBF-1*, *ACC* and *FASN*, were downregulated [13]. In fasted rodents, hepatic *SREBF-1* was reduced, leading to a decrease of fatty acid synthesis [32]. However, in the present study the lipogenic activity was not affected by the NEB in mid-lactation suggesting that not only energy balance, but also stage of lactation influences fatty acid synthesis.

Another factor involved in hepatic fatty acid metabolism is PPARα. PPARα is a nuclear receptor which regulates the expression of genes involved in peroxisomal and mitochondrial fatty acid β-oxidation [33]. PPARα plays an important role during periods of food deprivation by stimulation of the β-oxidation to cope with the increased hepatic supply of FFA [24]. During the deliberately induced NEB at 100 DIM, the expression of *PPARα* showed no change in RES cows. Previous studies with fasted mice [34] and dairy cows subjected to feed restriction leading to ketosis at the onset of lactation [13] reported an upregulation of hepatic *PPARα* by simultaneous high concentrations of plasma FFA. In contrast to the NEB in early lactation, the lack of change in the expression of *PPARα* during the induced NEB in mid-lactation might be explained by the lower plasma FFA concentration compared to early lactation [1].

PPARγ, a nuclear receptor implicated in adipogenesis, shows highest expression levels in adipose tissue and moderate levels in the liver [35]. In a study conducted with mice PPARγ was shown to regulate TAG homeostasis and to contribute to hepatic steatosis [36]. In the present study, no change in the hepatic mRNA abundance of *PPARγ* was detected during the NEB in mid-lactation. As previously suggested by van Dorland et al. [37], the unaltered *PPARγ* expression likely indicates that cows did not develop a fatty liver confirming findings of the present study [11].

Fat is an important milk component with TAG as major constituent and cholesterol representing a minor fraction [38,39]. The total cholesterol concentration in milk was not affected by the feed restriction in mid-lactation, but the cholesterol mass secreted in milk decreased in RES cows and tended to be lower compared to CON cows. This reduced cholesterol mass with a simultaneously decreased milk yield during feed restriction [1] explains the constant milk C concentration. Furthermore, in week 3 of feed restriction, the cholesterol concentration in the fat fraction of RES cows was reduced, but the milk fat mass in RES cows was not altered. Hence, the decreased cholesterol content in the milk fat in RES cows at week 3 can be explained by the lower milk cholesterol mass and the simultaneously unaltered fat mass.

A lower cholesterol content in the milk of underfed cows was previously reported by Precht [40] proposing a decreased activity of hepatic HMGCR during periods of undernourishment. However, in the present study the hepatic mRNA abundance of factors involved in cholesterol synthesis were not affected by the deliberately induced NEB. Thus, the reduced cholesterol mass in milk might be related to the simultaneously elevated hepatic demand of cholesterol for the formation of VLDL. Whether the reduced cholesterol mass in milk is causally related to the induced NEB or a consequence of the adapted liver metabolism remains to be elucidated.

## Conclusions

In mid-lactation, the cholesterol metabolism in dairy cows reacts differently to a NEB compared to early lactation. At approximately 100 DIM, the TAG derived from the re-esterification of mobilized FFA are exported from the liver by an enhanced formation of VLDL presumably to prevent hepatic steatosis. Important constituents of lipoproteins, such as cholesterol and PL, are sufficiently available in mid-lactation, thus a regulatory response on the mRNA expression level of genes involved in cholesterol synthesis is not observed. Contrary to early lactation, the decreased milk cholesterol content in mid-lactation might be a physiological adaptation of the liver to primarily provide cholesterol for the constitution of hepatic VLDL. However, the profound differences in the physiological response to the NEB at early compared to mid-lactation, might not only be due to metabolic differences resulting from the lactational stage, but also be a consequence of the extent and magnitude of the energy deficit provoking different regulatory processes in the liver.

## Supporting Information

S1 Dataset Experimental data of the feed-restriction trial. Data in columns provide the weekly data shown in the manuscript on production, metabolic, and endocrine data as well as data on hepatic mRNA abundance.
(XLS)

## References

[1] Gross J, van Dorland HA, Bruckmaier RM, Schwarz FJ. Performance and metabolic profile of dairy cows during a lactational and deliberately induced negative energy balance by feed restriction with subsequent realimentation. J Dairy Sci. 2011;94: 1820–1830. 10.3168/jds.2010-3707 21426971

[2] Drackley JK. ADSA Foundation Scholar Award. Biology of dairy cows during the transition period: the final frontier? J Dairy Sci. 1999;82: 2259–2273. 10575597 10.3168/jds.s0022-0302(99)75474-3

[3] Grummer RR. Etiology of lipid-related metabolic disorders in periparturient dairy cows. J Dairy Sci. 1993;76: 3882–3896. 8132893 10.3168/jds.S0022-0302(93)77729-2

[4] Kessler EC, Gross JJ, Bruckmaier RM, Albrecht C. Cholesterol metabolism, transport, and hepatic regulation in dairy cows during transition and early lactation. J Dairy Sci. 2014;97: 5481–5490. 10.3168/jds.2014-7926 24952770

[5] Gross J, van Dorland HA, Schwarz FJ, Bruckmaier RM. Endocrine changes and liver mRNA abundance of somatotropic axis and insulin system constituents during negative energy balance at different stages of lactation in dairy cows. J Dairy Sci. 2011;94: 3484–3494. 10.3168/jds.2011-4251 21700035

[6] Bjerre-Harpøth V, Friggens NC, Thorup VM, Larsen T, Damgaard BM, Ingvartsen KL, et al Metabolic and production profiles of dairy cows in response to decreased nutrient density to increase physiological imbalance at different stages of lactation. J Dairy Sci. 2012;95: 2362–2380. 10.3168/jds.2011-4419 22541465

[7] Friedewald WT, Levy RI, Fredrickson DS. Estimation of the concentration of low-density lipoprotein cholesterol in plasma, without use of the preparative ultracentrifuge. Clin Chem. 1972;18: 499–502. 4337382

[8] Paradkar MM, Irudayaraj J. Determination of cholesterol in dairy products using infrared techniques: 1. FTIR spectroscopy. Int J Dairy Technol. 2002;55:127–132.

[9] Kamelska AM, Pietrzak-Fiećko R, Bryl K. Variation of the cholesterol content in breast milk during 10 days collection at early stages of lactation. Acta Biochim Pol. 2012;59: 243–247. 22540113

[10] Hayirli A. The role of exogenous insulin in the complex of hepatic lipidosis and ketosis associated with insulin resistance phenomenon in postpartum dairy cows. Vet Res Commun. 2006; 30: 749–774. 17004039 10.1007/s11259-006-3320-6

[11] Gross JJ, Schwarz FJ, Eder K, van Dorland HA, Bruckmaier RM. Liver fat content and lipid metabolism in dairy cows during early lactation and during a mid-lactation feed restriction. J Dairy Sci. 2013;96: 5008–5017. 10.3168/jds.2012-6245 23746584

[12] Schlegel G, Ringseis R, Keller J, Schwarz FJ, Eder K. Changes in the expression of hepatic genes involved in cholesterol homeostasis in dairy cows in the transition period and at different stages of lactation. J Dairy Sci. 2012;95: 3826–3836. 10.3168/jds.2011-5221 22720938

[13] Loor JJ, Everts RE, Bionaz M, Dann HM, Morin DE, Oliveira R, et al Nutrition-induced ketosis alters metabolic and signaling gene networks in liver of periparturient dairy cows. Physiol Genomics. 2007;32: 105–116. 17925483 10.1152/physiolgenomics.00188.2007

[14] Ikonen E. Cellular cholesterol trafficking and compartmentalization. Nat Rev Mol Cell Biol. 2008;9: 125–138. 10.1038/nrm2336 18216769

[15] Van den Top AM, Geelen MJ, Wensing T, Wentink GH, Van ‘t Klooster AT, Beynen AC. Higher postpartum hepatic triacylglycerol concentrations in dairy cows with free rather than restricted access to feed during the dry period are associated with lower activities of hepatic glycerolphosphate acyltransferase. J Nutr. 1996;126: 76–85. 8558328 10.1093/jn/126.1.76

[16] Kulinski A, Rustaeus S, Vance JE. Microsomal triacylglycerol transfer protein is required for lumenal accretion of triacylglycerol not associated with ApoB, as well as for ApoB lipidation. J Biol Chem. 2002;277: 31516–31525. 12072432 10.1074/jbc.M202015200

[17] Bremmer DR, Trower SL, Bertics SJ, Besong S, Bernabucci U, Grummer RR. Etiology of fatty liver in dairy cattle: effects of nutritional and hormonal status on hepatic microsomal triglyceride transfer protein. J Dairy Sci. 2000;83: 2239–2251. 11049064 10.3168/jds.S0022-0302(00)75108-3

[18] Kang SW, Ahn EM, Cha YS. Changes in lipid and carnitine concentrations following repeated fasting-refeeding in mice. Nutr Res Pract. 2010;4: 477–485. 10.4162/nrp.2010.4.6.477 21286405 PMC3029788

[19] Nakagawa H, Oikawa S, Oohashi T, Katoh N. Decreased serum lecithin-cholesterol acyltranferase activity in spontaneous cases of fatty liver in cows. Vet Res Commun. 1997;21: 1–8. 9060137 10.1023/b:verc.0000009695.02015.20

[20] Pösö AR, Saukko TM, Tesfa AT, Lindberg L-A. Fat infiltration in liver and activity of lecithin:cholesterol acyltransferase in serum of dry and lactating dairy cows. Res Vet Sci. 2000;68: 169–173. 10756135 10.1053/rvsc.1999.0355

[21] Borggreve SE, de Vries R, Dullaart RPF. Alterations in high-density lipoprotein metabolism and reverse cholesterol transport in insulin resistance and type 2 diabetes mellitus: role of lipolytic enzymes, lecithin:cholesterol acyltransferase and lipid transfer proteins. Eur J Clin Invest. 2003;33: 1051–1069. 14636288 10.1111/j.1365-2362.2003.01263.x

[22] Jonas A, Sweeny SA, Herbert PN. Discoidal complexes of A and C apolipoproteins with lipids and their reactions with lecithin:cholesterol acyltransferase. J Biol Chem. 1984;259: 6369–6375. 6427214

[23] Tall AR, Krumholz S, Olivecrona T, Deckelbaum RJ. Plasma phospholipid transfer protein enhances transfer and exchange of phospholipids between very low density lipoproteins and high density lipoproteins during lipolysis. J Lipid Res. 1985;26: 842–851. 4031662

[24] Wolfbauer G, Albers JJ, Oram JF. Phospholipid transfer protein enhances removal of cellular cholesterol and phospholipids by high-density lipoprotein apolipoproteins. Biochim Biophys Acta. 1999;1439: 65–76. 10395966 10.1016/s1388-1981(99)00077-3

[25] Desvergne B, Michalik L, Wahli W. Transcriptional regulation of metabolism. Physiol Rev. 2006;86: 465–514. 16601267 10.1152/physrev.00025.2005

[26] Oram JF. HDL apolipoproteins and ABCA1: partners in the removal of excess cellular cholesterol. Arterioscler Thromb Vasc Biol. 2003;23: 720–727. 12615680 10.1161/01.ATV.0000054662.44688.9A

[27] Vaughan AM, Oram JF. ABCA1 and ABCG1 or ABCG4 act sequentially to remove cellular cholesterol and generate cholesterol-rich HDL. J Lipid Res. 2006;47: 2433–2443. 16902247 10.1194/jlr.M600218-JLR200

[28] Mani O, Sorensen MT, Sejrsen K, Bruckmaier RM, Albrecht C. Differential expression and localization of lipid transporters in the bovine mammary gland during the pregnancy-lactation cycle. J Dairy Sci. 2009;92: 3744–3756. 10.3168/jds.2009-2063 19620656

[29] Mani O, Körner M, Sorensen MT, Sejrsen K, Wotzkow C, Ontsouka CE, et al Expression, localization, and functional model of cholesterol transporters in lactating and nonlactating mammary tissues of murine, bovine, and human origin. Am J Physiol Regul Integr Comp Physiol. 2010;299: R642–R654. 10.1152/ajpregu.00723.2009 20445153

[30] Schultz JR, Tu H, Luk A, Repa JJ, Medina JC, Li L, et al Role of LXRs in control of lipogenesis. Genes Dev. 2000;14: 2831–2838. 11090131 10.1101/gad.850400 PMC317060

[31] Graber M, Kohler S, Kaufmann T, Doherr MG, Bruckmaier RM, van Dorland HA. A field study on characteristics and diversity of gene expression in the liver of dairy cows during the transition period. J Dairy Sci. 2010;93: 5200–5215. 10.3168/jds.2010-3265 20965335

[32] Horton JD, Shimomura I, Brown MS, Hammer RE, Goldstein JL, Shimano H. Activation of cholesterol synthesis in preference to fatty acid synthesis in liver and adipose tissue of transgenic mice overproducing sterol regulatory element-binding protein-2. J Clin. Invest. 1998;101: 2331–2339. 9616204 10.1172/JCI2961 PMC508822

[33] Mandard S, Müller M, Kersten S. Peroxisome proliferator-activated receptor alpha target genes. Cell Mol Life Sci. 2004:61: 393–416. 14999402 10.1007/s00018-003-3216-3 PMC11138883

[34] Kersten S, Seydoux J, Peters JM, Gonzalez FJ, Desvergne B, Wahli W. Peroxisome proliferator-activated receptor alpha mediates the adaptive response to fasting. J Clin Invest. 1999;103: 1489–1498. 10359558 10.1172/JCI6223 PMC408372

[35] Desvergne B, Wahli W. Peroxisome proliferator-activated receptors: nuclear control of metabolism. Endocr Rev. 1999;20: 649–688. 10529898 10.1210/edrv.20.5.0380

[36] Gavrilova O, Haluzik M, Matsusue K, Cutson JJ, Johnson L, Dietz KR, et al Liver peroxisome proliferator-activated receptor gamma contributes to hepatic steatosis, triglyceride clearance, and regulation of body fat mass. J Biol Chem. 2003;278: 34268–34276. 12805374 10.1074/jbc.M300043200

[37] Van Dorland HA, Richter S, Morel I, Doherr MG, Castro N, Bruckmaier RM. Variation in hepatic regulation of metabolism during the dry period and in early lactation in dairy cows. J Dairy Sci. 2009;92: 1924–1940. 10.3168/jds.2008-1454 19389950

[38] Bitman J, Wood DL. Changes in milk fat phospholipids during lactation. J Dairy Sci. 1990;73: 1208–1216. 2365882 10.3168/jds.S0022-0302(90)78784-X

[39] Ontsouka EC, Albrecht C. Cholesterol transport and regulation in the mammary gland. J Mammary Gland Biol Neoplasia. 2014;19: 43–58. 10.1007/s10911-014-9316-x 24510467

[40] Precht D. Cholesterol content in European bovine milk fats. Nahrung. 2001;45: 2–8. 11253633 10.1002/1521-3803(20010101)45:1<2::AID-FOOD2>3.0.CO;2-5

